# Baseline Detection of Honey Bee Viruses and *Nosema ceranae* in *Apis* Species from Cambodia and Laos

**DOI:** 10.3390/insects17090956

**Published:** 2026-09-14

**Authors:** Tial C. Ling, Thunyarat Chantaphanwattana, Patcharin Phokasem, Chainarong Sinpoo, Chhouk Chheang, Viengkhone Vannachak, Yulong Guo, Shaokang Huang, Eui-Joon Kil, Terd Disayathanoowat

**Affiliations:** 1 Department of Biology, Faculty of Science, Chiang Mai University, Chiang Mai 50200, Thailand; tialcungling@gmail.com or tcling@ohio.edu (T.C.L.); thunyarat.chan@gmail.com (T.C.); patcharin.ph@cmu.ac.th (P.P.); chainarong.s@cmu.ac.th (C.S.); 2 Department of Environmental and Plant Biology, Ohio University, 22 Richland Ave., 401 Porter Hall, Athens, OH 45701, USA; 3 Office of Research Administration, Chiang Mai University, Chiang Mai 50200, Thailand; 4 Research Center of Deep Technology in Beekeeping and Bee Products for Sustainable Development Goals (SMART BEE SDGs), Chiang Mai University, Chiang Mai 50200, Thailand; 5 Department of Agronomy, Faculty of Agriculture and Food Processing, National Meanchey University, Serei Saophoan 010600, Banteay Meanchey Province, Cambodia; chheang.chhouk.mcu@moeys.gov.kh; 6 Department of Biology, Faculty of Natural Sciences, National University of Laos, Vientiane 7322, Laos; v.vannachak@nuol.edu.la; 7 Yunnan Provincial Engineering and Research Center for Sustainable Utilization of Honey Bee Resources, Eastern Bee Research Institute, College of Animal Science and Technology, Yunnan Agricultural University, Kunming 650201, China; guoyulong123@hotmail.com; 8 College of Bee Science and Biomedicine, Fujian Agriculture and Forestry University, Fuzhou 350002, China; skhuang@fafu.edu.cn; 9 Department of Plant Medicals, Gyeongkuk National University, Andong 36729, Republic of Korea; viruskil@gknu.ac.kr

**Keywords:** *Apis* species, honey bee pathogens, *Nosema ceranae*, pathogen transmission, Southeast Asia, viruses

## Abstract

Honey bees provide essential pollination services in natural and agricultural ecosystems, yet little is known about honey bee-associated pathogens in Cambodia and Laos. Here, we screened 53 colonies from 10 sites, including *Apis cerana* (*n* = 28), *A*. *mellifera* (*n* = 18), *A*. *dorsata* (*n* = 4), and *A*. *florea* (*n* = 3), for common honey bee viruses and the microsporidian *Nosema ceranae* using PCR and RT-PCR assays. Six viruses—acute bee paralysis virus, black queen cell virus, deformed wing virus type A, Israeli acute paralysis virus, Kashmir bee virus, and Lake Sinai virus—and *N*. *ceranae* were detected in *A*. *cerana* and *A*. *mellifera*, with overall low detection frequencies across sites. No targets were detected in *A*. *dorsata* or *A*. *florea*; however, the limited sample sizes for these species restrict interpretation of absence. Partial gene fragment sequences showed similarity to previously reported Asian isolates, indicating consistency with known regional viral diversity. These findings provide baseline detection data for honey bee-associated pathogens in Cambodia and Laos and support the need for expanded sampling across host species and seasons to better characterize pathogen occurrence in the region.

## Simple Summary

Honey bees are important pollinators of wild plants and crops, but little is known about the viruses and microscopic parasites associated with honey bees in Cambodia and Laos. We examined 53 colonies from 10 locations to determine whether commonly reported honey bee viruses and a microscopic parasite called *Nosema ceranae* were present. Six viruses and *N*. *ceranae* were detected, generally at low frequencies. These organisms were found only in colonies of the eastern honey bee and the western honey bee. None were detected in the small numbers of giant honey bee and dwarf honey bee colonies examined, although the limited sampling means that their absence cannot be confirmed. Genetic comparisons showed that the detected viruses and parasite were similar to previously reported sequences from Asia and other regions. These findings provide important baseline information on honey bee-associated pathogens in the sampled areas of Cambodia and Laos. The results can support future monitoring, help identify changes in pathogen occurrence, and guide research aimed at protecting honey bee health, beekeeping, crop pollination, and wild plant reproduction in the Mekong region.

## 1. Introduction

Honey has been valued for millennia for its nutritional, medicinal, and cultural importance [1]. Beyond honey production, honey bees (*Apis* species) play a central role in pollination, supporting both wild plant reproduction and agricultural productivity [2,3,4,5]. While concerns about pollinator health have received considerable attention, long-term assessments of managed honey bee populations do not indicate a uniform global decline. Instead, colony numbers have increased worldwide, although parts of Europe and North America have experienced elevated and sometimes unexplained colony losses [6,7]. These losses frequently occur during winter and are influenced by interacting biological, environmental, management, and socio-economic factors [8,9,10]. These regional differences highlight the importance of evaluating honey bee health within specific ecological and management contexts.

Among biotic stressors, the ectoparasitic mite *Varroa destructor* has significantly altered honey bee disease dynamics in many regions. In addition to its direct effects on colony fitness, *Varroa* acts as an efficient vector and amplifier of several RNA viruses [11,12,13]. Deformed wing virus (DWV) is among the most widely detected viruses associated with *Varroa* infestation, and the DWV complex comprises multiple master variants with distinct evolutionary histories and epidemiological patterns [12]. Historical analyses have further shown that DWV-related viruses were present earlier than often assumed; Egypt bee virus (EBV), detected in 1977, has since been recognized as a distinct DWV master strain (DWV-D), underscoring the long-standing diversity within this viral complex [14].

Microsporidia of the genus *Nosema*, particularly *N*. *ceranae*, are also commonly reported in honey bee colonies [15,16]. However, the relationship between *N*. *ceranae* infection and colony mortality remains debated. Several regional and longitudinal studies have found limited or inconsistent associations between *Nosema* infection status and colony losses [17,18,19,20], and long-term monitoring data suggest that, although statistical associations may occur in large datasets, the biological impact at the colony level may be modest relative to dominant stressors such as *Varroa* infestation [21]. Accordingly, pathogen detection alone does not necessarily indicate colony decline or impaired health.

In Asia, research on honey bee pathogens has expanded substantially in recent years, particularly in regions where introduced *Apis mellifera* coexists with native species such as *A*. *cerana*. Viral detection frequencies can differ between these hosts and across seasons [22], and several viruses, including DWV and black queen cell virus (BQCV), have been detected in both species where they occur sympatrically [23]. Surveys in Thailand and neighboring regions have also documented ABPV, BQCV, Israeli acute paralysis virus (IAPV), and Kashmir bee virus (KBV) [24,25]. Their occurrence can vary with host species, season, location, and colony-management context [22,23,24,25]. Published data from Cambodia and Laos nevertheless remain scarce, although managed and wild *A*. *cerana*, *A*. *mellifera*, *A*. *dorsata*, and *A*. *florea* occur in close proximity within the Mekong region.

The present study therefore screened colonies from multiple sites in Cambodia and Laos for selected honey bee viruses and *Nosema* species using targeted molecular assays. The objective was to document the occurrence of these agents in sampled colonies and, where sequence data were available, to compare partial gene fragments with reference strains reported from other regions. Because sample sizes for some species were limited and colony-level health parameters were not assessed, the findings are presented as preliminary detection data. Non-detection should not be interpreted as evidence of pathogen absence, nor do the data allow inference regarding transmission dynamics or population-level impacts. Rather, the results provide a baseline reference to support future, more comprehensive surveys in the region.

## 2. Materials and Methods

### 2.1. Honey Bee Sample Collection

**Figure 1:**
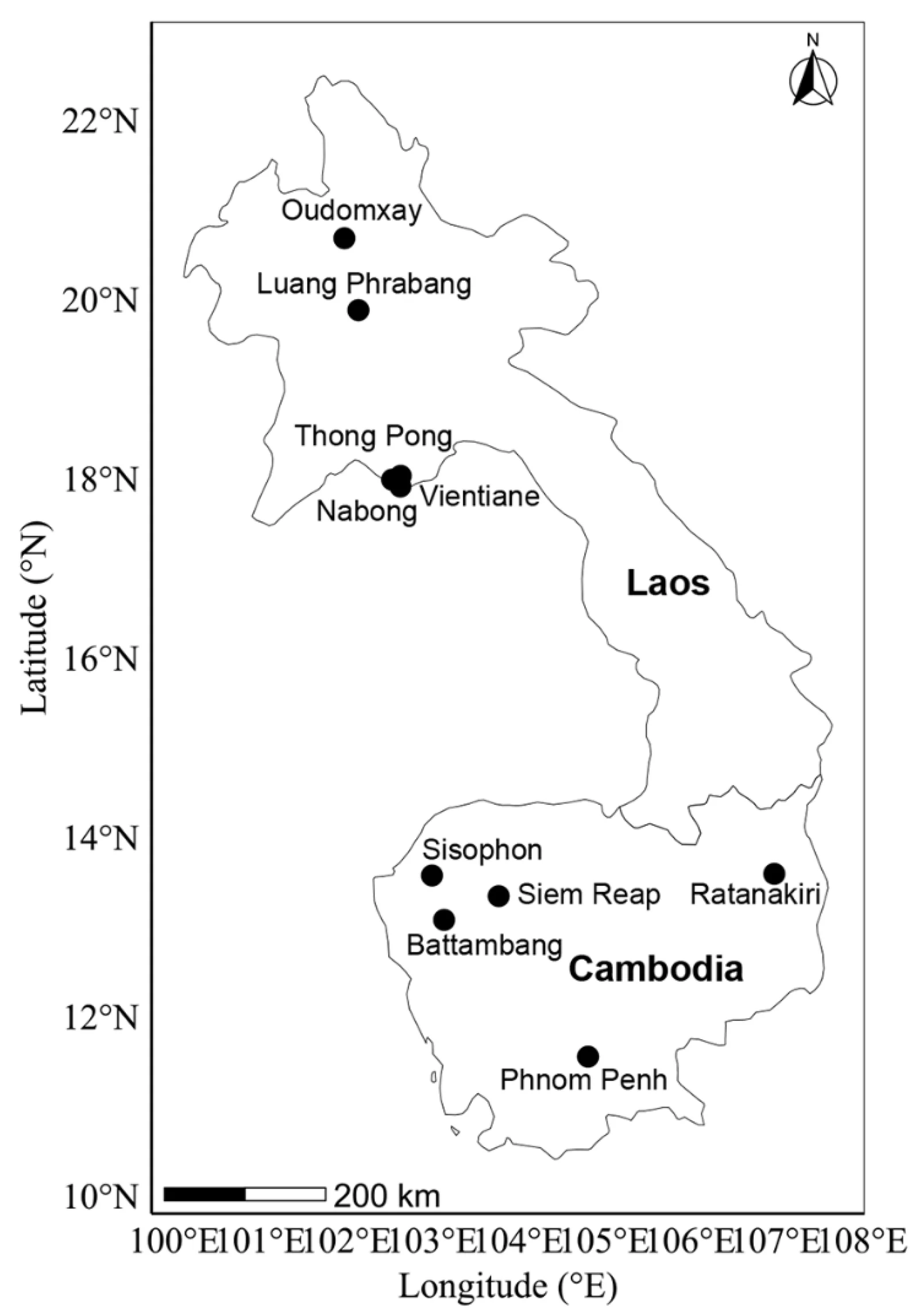
Sampling locations of honey bee colonies in Cambodia and Laos. Colonies were sampled from five sites in Cambodia: Sisophon, Battambang, Siem Reap, Phnom Penh, and Ratanakiri, and five sites in Laos: Oudomxay, Vientiane, Luang Prabang, Nabong, and Thong Pong. Sampling was conducted in Cambodia in May 2022 and July 2023, and in Laos in May 2022 and March 2023. Detailed colony numbers by site and species are provided in Table S1; pathogen-specific detection frequencies and viral detection patterns are summarized in Section, Figure 3, and Table 1.

A total of 53 colonies were sampled from five sites in Cambodia (Sisophon, Battambang, Siem Reap, Phnom Penh, and Ratanakiri) and five sites in Laos (Oudomxay, Vientiane, Luang Prabang, Nabong, and Thong Pong; Figure 1; Table S1). The sample comprised *A*. *cerana* (*n* = 28), *A*. *mellifera* (*n* = 18), *A*. *dorsata* (*n* = 4), and *A*. *florea* (*n* = 3). Cambodia contributed 30 colonies (10 *A*. *cerana*, 15 *A*. *mellifera*, 3 *A*. *dorsata*, and 2 *A*. *florea*), whereas Laos contributed 23 colonies (18 *A*. *cerana*, 3 *A*. *mellifera*, 1 *A*. *dorsata*, and 1 *A*. *florea*). Based on the geographic coordinates provided in Table S1, the approximate straight-line distances were 13.3 km between Vientiane and Nabong, 13.3 km between Vientiane and Thong Pong, and 11.6 km between Nabong and Thong Pong. Detailed geographic coordinates and colony counts by species and site are provided in Table S1.

**Table 1:** Frequencies of single-, dual-, and triple-virus detection patterns in *Apis* colonies sampled in Cambodia (*n* = 30) and Laos (*n* = 23). Values are presented as percentages, with the number of colonies exhibiting each virus or virus combination relative to the total number sampled shown in parentheses.

Country	Detection Category	Virus or Virus Combination	Detection Frequency
Cambodia	Single-virus detection	DWV-A	10.0% (3/30)
	Dual-virus detection	ABPV, DWV-A	3.3% (1/30)
Laos	Single-virus detection	BQCV	4.3% (1/23)
	Dual-virus detection	ABPV, IAPV	4.3% (1/23)
	Dual-virus detection	BQCV, LSV	8.7% (2/23)
	Triple-virus detection	ABPV, IAPV, KBV	4.3% (1/23)

Samples were obtained from managed, semi-managed, and wild colonies. Semi-managed *A*. *cerana* colonies occupied beekeeper-provided hive boxes but received minimal intervention: no syrup or pollen-patty feeding, queen replacement, or *Varroa* treatment was provided, and honey was harvested once before the colonies typically absconded. Information on colony age depended on management status. For managed colonies, age was estimated from beekeeper records, primarily annual queen-replacement records; comparable age information was unavailable for semi-managed and wild colonies.

From each colony, at least 50 worker honey bees were collected into sterile, labeled 50 mL conical tubes. Workers were collected from brood frames and comb surfaces where feasible to obtain colony-representative samples. Sampling was conducted during periods of high flowering and foraging activity in the region. Colonies in Cambodia were sampled in May 2022 and July 2023, whereas colonies in Laos were sampled in May 2022 and March 2023. The collection of at least 50 workers per colony was used to standardize laboratory processing across species. Differences in colony size among species were considered when interpreting detection and non-detection outcomes. Clinical signs were not evaluated using a predefined or standardized colony-health protocol. Consequently, pathogen-positive colonies could not be retrospectively classified as symptomatic or asymptomatic. In addition, the original study was designed as a targeted molecular detection survey rather than an assessment of relationships between pathogen occurrence and colony health. Standardized colony-strength variables, including adult bee population, brood area, and the number of frames occupied by bees, were not recorded. Consequently, the colonies could not be retrospectively categorized by strength using standardized colony-population measures comparable to those reported by Faurot-Daniels et al. [26] and Gonzalez et al. [27]. *Varroa destructor* infestation was not assessed as part of the original field-sampling protocol. Therefore, possible relationships between mite infestation and viral detection, particularly DWV-A detection, could not be evaluated.

Honey bee samples were preserved in RNAlater (Invitrogen, Vilnius, Lithuania) after collection to stabilize nucleic acids until transport. Because samples were collected from different countries, same-day transport to the laboratory was not feasible. Samples were transported under chilled conditions in insulated containers to the laboratory at Chiang Mai University, Thailand (18.8016° N, 98.9560° E, 336 m above sea level). Upon arrival, samples were stored at −80 °C until further processing.

### 2.2. DNA, RNA Extraction, and cDNA Synthesis

For each colony, pooled workers were homogenized under liquid nitrogen with a sterile mortar and pestle, and the homogenate was divided for separate DNA and RNA extraction. Genomic DNA was extracted with the PureLink™ Genomic DNA Mini Kit (Invitrogen, Carlsbad, CA, USA) according to the manufacturer’s protocol and stored at −20 °C until use. Total RNA was extracted from a separate portion of each colony homogenate using TRIzol reagent (Invitrogen, Carlsband, CA, USA) according to the manufacturer’s instructions. RNA was treated with DNase I at 37 °C for 1 h, followed by enzyme inactivation at 75 °C for 10 min, diluted to 100 μL with nuclease-free water, and stored at −20 °C until reverse transcription.

Complementary DNA (cDNA) was synthesized from total RNA with the Tetro cDNA Synthesis Kit (Bioline, Memphis, TN, USA) using oligo (dT)18 primers and random hexamers. Reverse transcription was performed at 25 °C for 10 min, followed by 45 °C for 30 min, and enzyme inactivation at 85 °C for 5 min. The cDNA was stored at −20 °C until use in the two-step RT-PCR assays described in Section 2.4.

### 2.3. DNA Barcoding and COI Sequence Analysis

Honey bee species were initially identified from morphology. Species identity was confirmed by PCR amplification of the mitochondrial cytochrome c oxidase subunit I (COI) barcode fragment using primers LCO1490 (5′-GGT CAA CAA ATC ATA AAG ATA TTGG-3′) and HCO2198 (5′-TAA ACT TCA GGG TGA CCA AAAAAT CA-3′) following Folmer et al. [28]. Each 25 µL reaction contained 2.5 µL 10× PCR buffer, 0.75 µL of 20 mM MgCl_2_, 0.5 µL 10 mM dNTP mix, 1.25 µL of each primer (forward and reverse), 0.1 µL of 5 U/µL Taq DNA polymerase (Invitrogen, Carlsbad, CA, USA), 17.65 µL DNase- and RNase-free water, and 1 µL genomic DNA from the corresponding colony. Thermal cycling comprised an initial denaturation at 94 °C for 3 min, followed by 35 cycles of 94 °C for 30 s, 50 °C for 30 s, and 72 °C for 1 min, with a final extension at 72 °C for 10 min. Products were visualized on 1.5% agarose gels. Selected COI amplicons were purified and Sanger sequenced by Macrogen (Seoul, Republic of Korea). Sequences were edited in BioEdit v7.2.5.0, compared with GenBank records using BLAST program (available from the National Center for Biotechnology Information (NCBI)), aligned with representative references, and analyzed using MEGA X and PhyML 3.0 with 10,000 bootstrap replicates. COI accession numbers and sample metadata are provided in Table S3.

### 2.4. Pathogen Screening via PCR and RT-PCR

Colonies were screened for 10 RNA viruses and six DNA-based pathogens (Tables S2 and S4). The RNA–virus panel comprised ABPV, BQCV, CBPV, DWV, DWV-B, IAPV, LSV, KBV, SBV, and SBPV. These targets were screened via conventional PCR using colony-derived cDNA as the template after the reverse-transcription step described in Section 2.2 (two-step RT-PCR). The DNA–pathogen panel comprised *N*. *apis*, *N*. *bombi*, *N*. *ceranae*, *Paenibacillus larvae*, *Ascosphaera apis*, and *Crithidia bombi*; these targets were screened via conventional PCR using genomic DNA extracted from the corresponding colony homogenate. Thus, RNA–virus assays used cDNA, whereas DNA–pathogen assays used genomic DNA.

For viral targets, amplification comprised 94 °C for 7 min, 40 cycles of 94 °C for 45 s; annealing for 1 min at 55 °C (BQCV, CBPV, DWV, DWV-B, KBV, and SBV), 52 °C (ABPV, IAPV, and SBPV), or 60 °C (LSV); and 72 °C for 1 min, followed by 72 °C for 7 min. DNA-based pathogens were amplified using the target-specific conditions described in the corresponding source references listed in Table S2. Amplicons were separated on 1.5% agarose gels and visualized under UV illumination with a 100 bp DNA ladder (GeneDireX, Taoyuan, Taiwan). Each PCR run included a no-template control and, when available, a previously confirmed target-positive control. The β-actin assay was used as an internal amplification control. Primer sequences, expected amplicon sizes, and source references are provided in Table S2.

### 2.5. Pathogen Amplicon Sequencing and Phylogenetic Analysis

Selected target-positive viral and *N*. *ceranae* amplicons were purified and Sanger sequenced by Macrogen (Seoul, Republic of Korea). Sequence chromatograms were edited and assembled in BioEdit v7.2.5.0 (Ibis Biosciences, Carlsbad, CA, USA), and target identity was assessed using BLAST searches against the NCBI nucleotide database. Accession numbers and associated sample information for the viral and *N*. *ceranae* sequences generated in this study are provided in Table S3.

For each detected virus and *N*. *ceranae*, partial sequences were aligned separately with representative GenBank reference sequences. Maximum-likelihood trees were constructed from the corresponding partial gene alignments using MEGA X and PhyML 3.0 with 10,000 bootstrap replicates. These analyses were used to confirm target identity and compare each amplified region with available reference sequences.

### 2.6. Statistical Analysis

Aggregated colony-level detection frequency was calculated separately for each screened pathogen and country as the number of colonies positive for that pathogen divided by the total number of colonies sampled in the country. These pooled country-level frequencies combine locations and collection dates and are therefore not point-prevalence estimates for individual sampling events. Viral co-detection was summarized separately as the numbers and percentages of colonies with single-, dual-, or triple-virus detections; *N*. *ceranae* was excluded from these viral-pattern categories. No inferential statistical comparisons were performed because sample sizes were small and uneven. Calculations and graphical summaries were produced in R version 4.3.1 [29].

## 3. Results

### 3.1. DNA Barcoding

**Figure 2:**
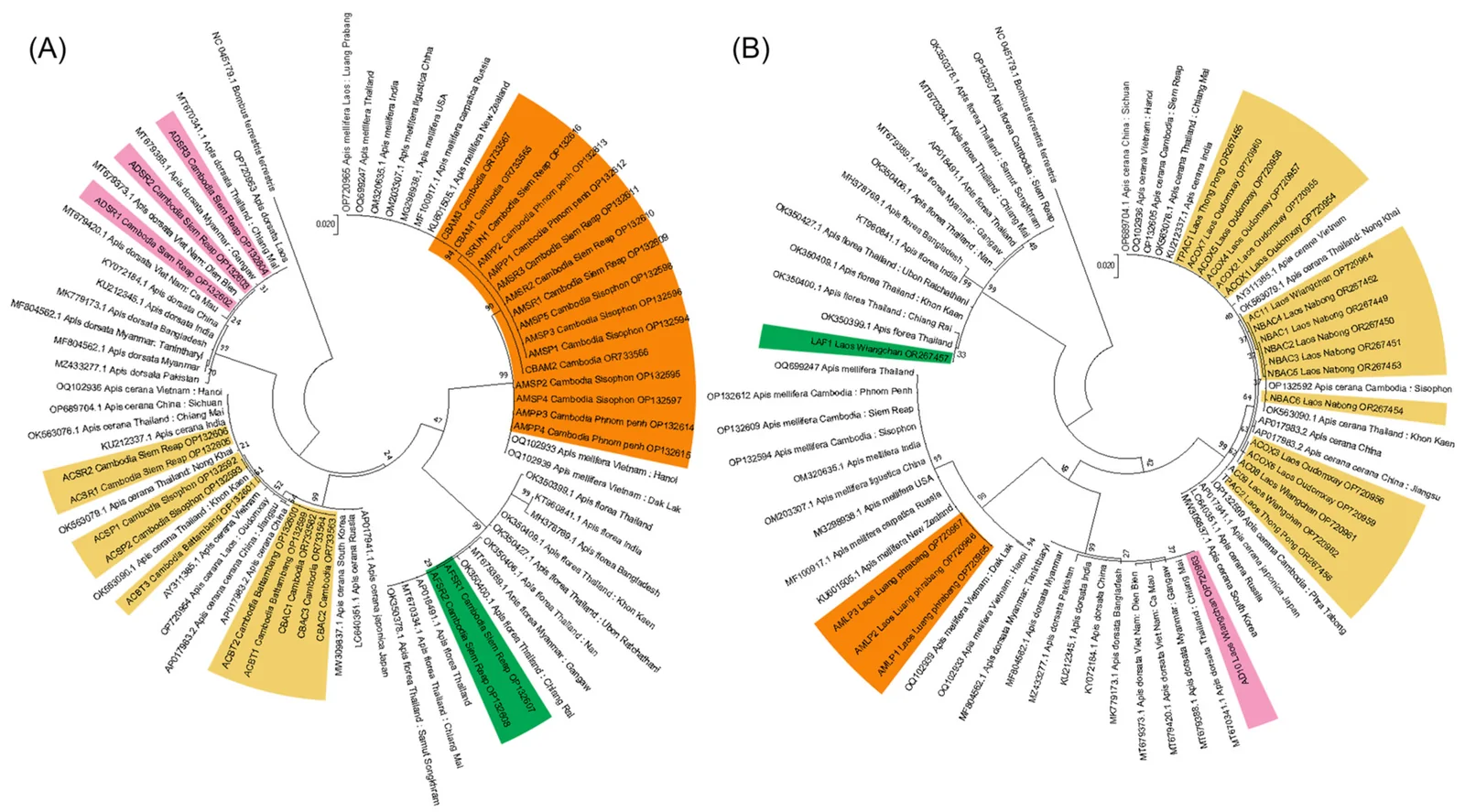
Cytochrome c oxidase-based maximum-likelihood trees of *Apis* colonies sampled in (**A**) Cambodia and (**B**) Laos. Sequences generated in this study are identified by their field sample codes and colored by species: *A*. *cerana* (yellow), *A*. *dorsata* (pink), *A*. *florea* (green), and *A*. *mellifera* (orange); reference sequences are labeled with GenBank accession numbers. The country, site, species, and accession associated with each study code are listed in Table S3. Bootstrap support values are shown at nodes, and scale bars indicate nucleotide substitutions per site.

COI barcode amplification was successful for all 53 colonies (100%). Sequence comparisons confirmed every field identification, with no discrepancy between morphological and molecular assignments (Figure 2). The resulting sequences represented *A*. *cerana*, *A*. *mellifera*, *A*. *dorsata*, and *A*. *florea*; accession numbers and sample metadata are provided in Table S3.

### 3.2. Pathogen Detection Frequencies and Viral Detection Patterns

**Figure 3:**
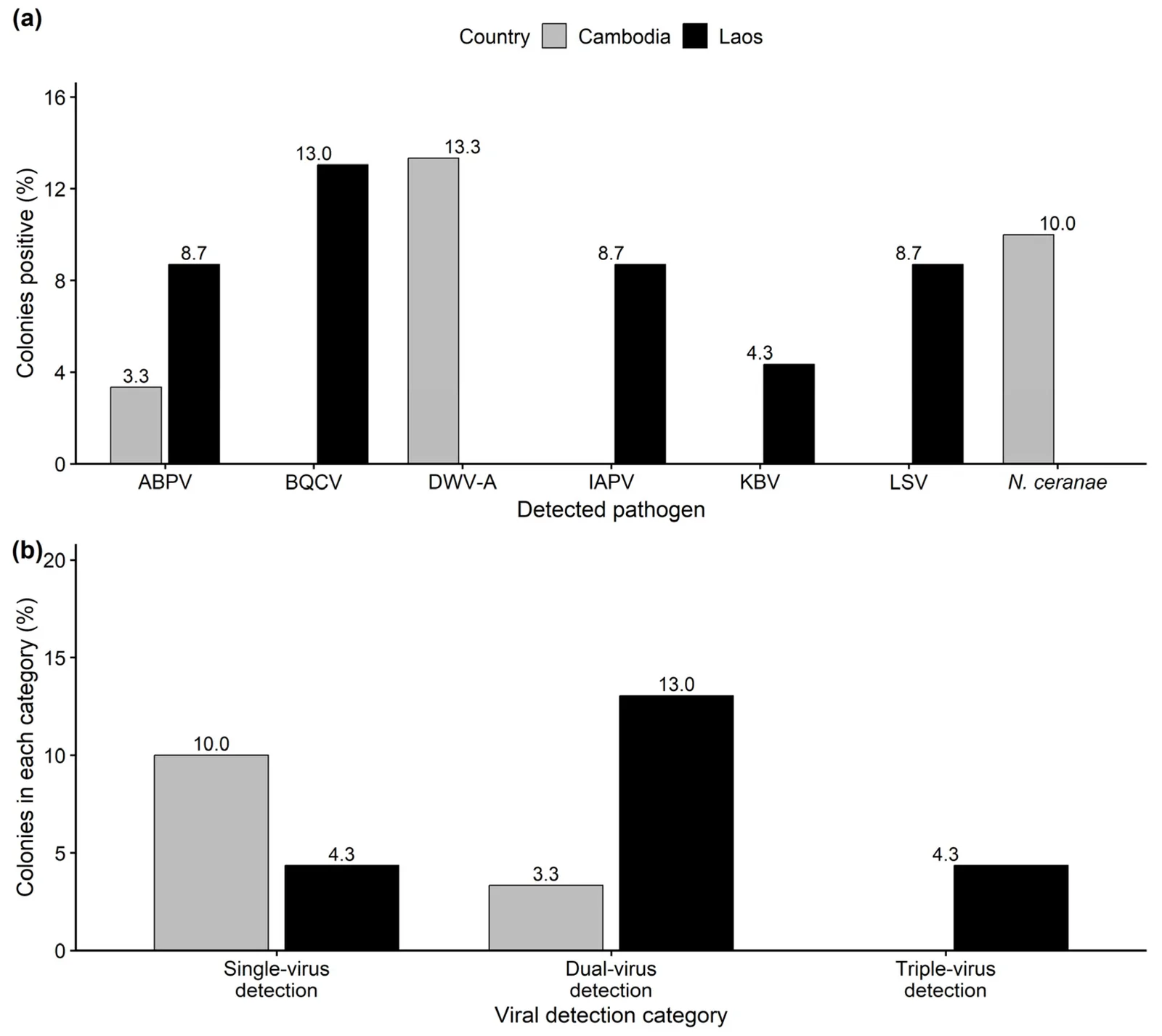
Pathogen-specific detection frequencies and positive viral detection patterns in colonies sampled from Cambodia and Laos. (**a**) Percentages of colonies positive for each detected target: acute bee paralysis virus (ABPV), black queen cell virus (BQCV), deformed wing virus type A (DWV-A), Israeli acute paralysis virus (IAPV), Kashmir bee virus (KBV), Lake Sinai virus (LSV), and *Nosema ceranae* (NC). (**b**) Percentages of colonies with single-, dual-, or triple-virus detections. *N*. *ceranae* was included in panel (**a**) but excluded from the viral co-detection pattern categories in panel (**b**). Country denominators were 30 colonies for Cambodia and 23 colonies for Laos.

Seven targets were detected: ABPV, BQCV, DWV-A, IAPV, LSV, KBV, and *N*. *ceranae*. All positive detections occurred in *A*. *cerana* or *A*. *mellifera*. Pathogen-specific country frequencies are shown in Figure 3a, and colony-level results for all 10 RNA viruses and six DNA-based pathogens are provided in Table S4.

Table 1 summarizes the positive viral detection patterns. Cambodia had three DWV-A-only colonies and one colony with ABPV and DWV-A. Laos had one BQCV-only colony, one colony with ABPV and IAPV, two colonies with BQCV and LSV, and one colony with ABPV, IAPV, and KBV.

Multiple-virus detections occurred in four of 23 colonies from Laos (17.4%) and one of 30 colonies from Cambodia (3.3%). Among the Laos samples, these comprised two *A*. *cerana* colonies and two *A*. *mellifera* colonies; the Cambodian dual detection occurred in *A*. *mellifera*.

### 3.3. Sequence Comparison and Fragment-Based Phylogenetic Placement

**Figure 4:**
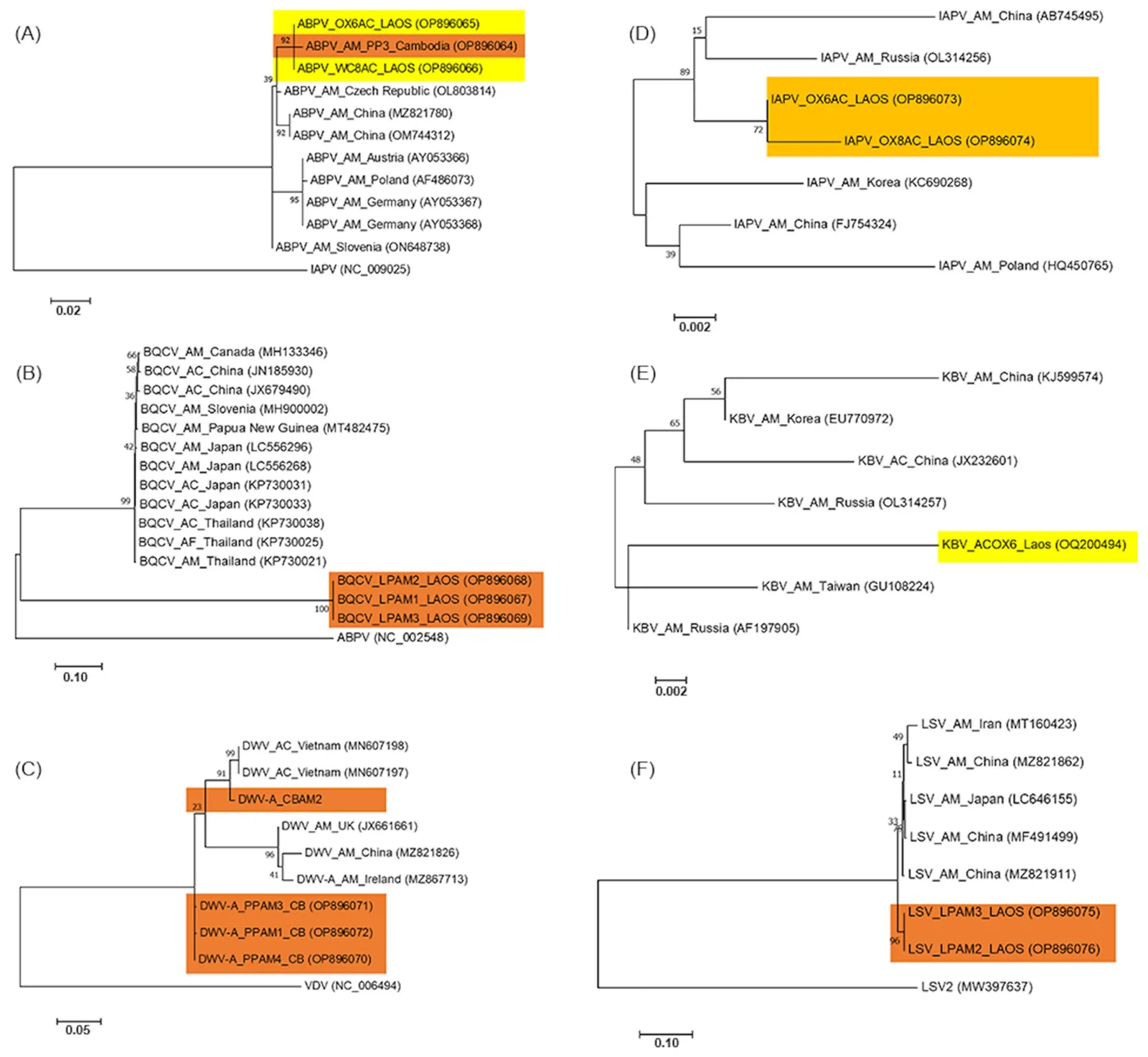
Maximum-likelihood phylogenetic trees based on partial PCR amplicon sequences of viruses detected in *Apis* species from Cambodia and Laos. Sequences generated in this study retain their field sample codes and are highlighted according to host species: *A*. *cerana* in yellow and *A*. *mellifera* in orange. Reference sequences are shown in black text and identified by their GenBank accession numbers. The country, site, host species, and accession number corresponding to each study-generated field code are provided in Table S3. The viruses analyzed include (**A**) acute bee paralysis virus (ABPV), (**B**) black queen cell virus (BQCV), (**C**) deformed wing virus type A (DWV-A), (**D**) Israeli acute paralysis virus (IAPV), (**E**) Kashmir bee virus (KBV), and (**F**) Lake Sinai virus (LSV). Bootstrap values >70% are shown at nodes. Scale bars indicate nucleotide substitutions per site. Trees are based on partial gene fragments and are presented to illustrate sequence similarity relative to reference strains.

**Figure 5:**
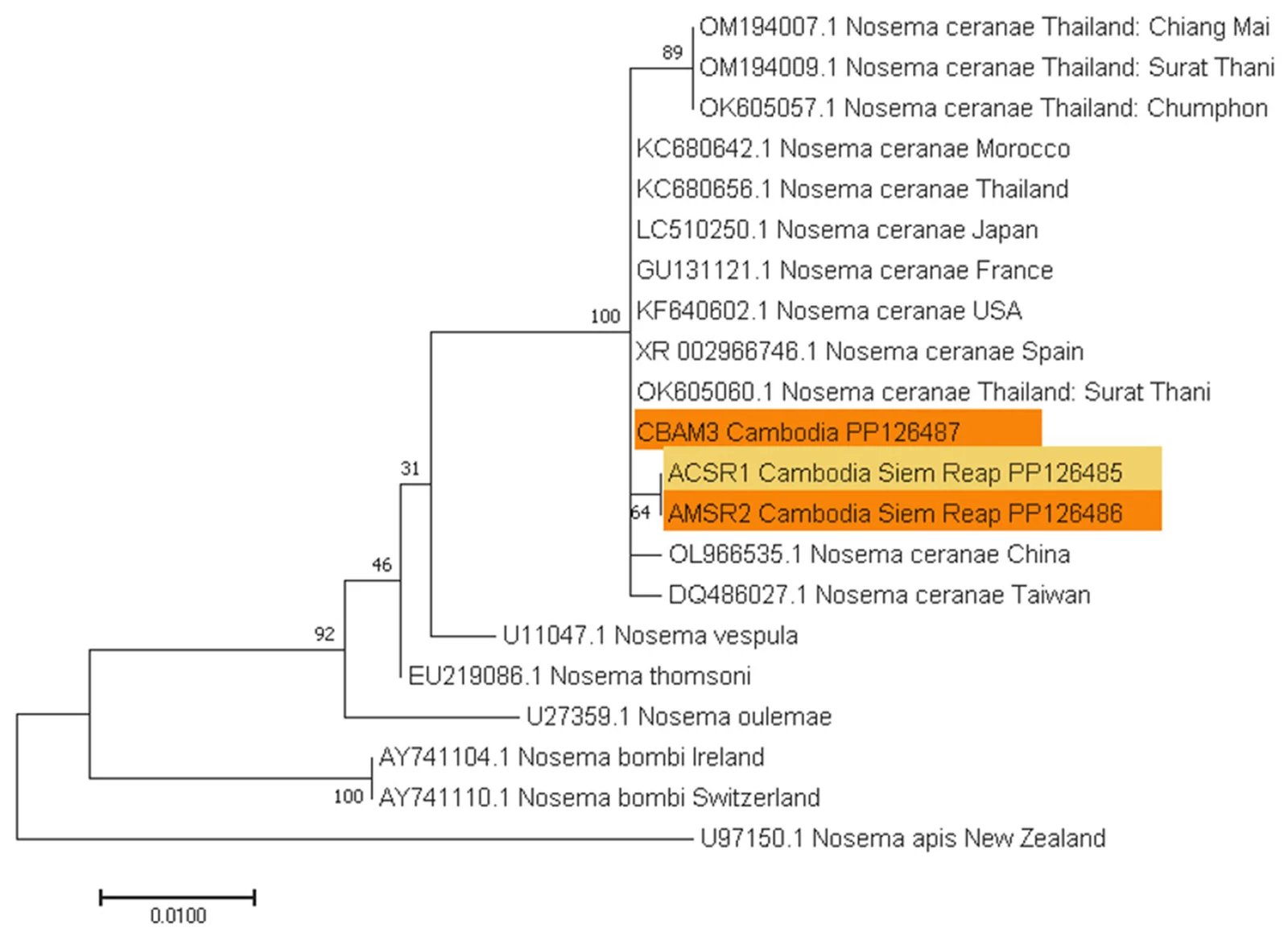
Maximum-likelihood phylogenetic tree based on partial gene sequences of *Nosema ceranae* detected in *A*. *cerana* (yellow) and *A*. *mellifera* (orange) from Cambodia. Sequences generated in this study retain their field sample codes, whereas reference sequences are identified by their GenBank accession numbers. The site, host species, and accession number corresponding to each study-generated field code are provided in Table S3. Reference sequences from other countries are included for comparison, with *N*. *apis* used as an outgroup. Bootstrap values (>70%) are shown at nodes. The scale bar represents nucleotide substitutions per site. The tree is based on a partial amplified region and reflects sequence similarity within the analyzed fragment.

Sanger sequencing of selected target-positive amplicons confirmed the identities of ABPV, BQCV, DWV-A, IAPV, KBV, LSV, and *N*. *ceranae*. The corresponding partial sequences and accession numbers are listed in Table S3. The resulting target-specific phylogenetic placements are presented in Figure 4 and Figure 5.

For ABPV, sequences from Laos (*A*. *cerana*) and Cambodia (*A*. *mellifera*) grouped with reference sequences previously reported from China and the Czech Republic (Figure 4A). BQCV sequences obtained from *A*. *mellifera* in Laos grouped with Asian reference sequences and formed a separate branch within the analyzed fragment (Figure 4B). The DWV primers were used for the detection of DWV, and variant identification was subsequently supported by Sanger sequencing and phylogenetic analysis. DWV-A was detected only in *A. mellifera* from Cambodia. To further verify the identity of the PCR-positive samples, three DWV-A-positive amplicons were subjected to Sanger sequencing. The resulting chromatograms were clear and unambiguous, with no evidence of mixed nucleotide peaks. Sequence comparison with reference DWV sequences in GenBank and phylogenetic analysis showed that all three sequences clustered with reference DWV-A sequences and were distinct from the DWV-B/VDV-1 reference sequence within the analyzed region (Figure 4C). The sequences from Phnom Penh and Ratanakiri showed minor nucleotide variation within the amplified region but clustered with previously reported DWV-A sequences from Vietnam and China (Figure 4C). IAPV sequences from *A*. *cerana* in Laos grouped with reference sequences from China, Russia, Poland, and Korea (Figure 4D). The KBV sequence from Laos grouped with previously reported Asian KBV sequences (Figure 4E). LSV sequences detected in *A*. *mellifera* from Laos clustered with sequences previously assigned to the LSV4 group based on similarity within the amplified region (Figure 4F).

Phylogenetic analysis of *N*. *ceranae* partial sequences confirmed their placement within the *N*. *ceranae* clade and separation from other *Nosema* species (Figure 5). Identical sequences were detected in *A*. *cerana* and *A*. *mellifera* from Siem Reap in Cambodia. Within the analyzed fragment, Cambodian sequences grouped with reference sequences reported from China, Taiwan, Morocco, Thailand, Japan, France, and the USA. Because analyses were based on partial PCR amplicons, the resulting trees are interpreted as indicative of sequence similarity within the amplified regions rather than definitive evolutionary or transmission relationships.

## 4. Discussion

To our knowledge, this study provides the first molecular survey of selected honey bee-associated viruses and *N*. *ceranae* across managed, semi-managed, and wild *Apis* colonies from Cambodia and Laos. Six RNA viruses (ABPV, BQCV, DWV-A, IAPV, LSV, and KBV) together with *N*. *ceranae* were detected, establishing a baseline for pathogen occurrence in this poorly studied part of the Mekong region.

### 4.1. Pathogen Occurrence in the Sampled Colonies

The detected viruses are established components of managed honey bee pathogen assemblages in Asia and elsewhere [24,25,30]. Their occurrence in both *A*. *cerana* and *A*. *mellifera* is consistent with the broad host ranges reported for many bee-associated viruses [22,23,31]. These species accounted for 46 of 53 colonies and included the managed and semi-managed colonies, providing greater detection opportunity than the seven *A*. *dorsata* and *A*. *florea* colonies.

Management may contribute to these patterns by altering colony density, movement, resource sharing, and parasite exposure, although management categories could not be compared formally in this dataset. DWV-A occurred only in Cambodian *A*. *mellifera* colonies, a pattern of interest because *V*. *destructor* can amplify DWV in managed *A*. *mellifera* populations [11,12,13]. Conversely, ABPV occurred in both countries and both principal host species, while BQCV, IAPV, LSV, and KBV were detected only in Laos. These observations identify useful targets for future surveillance of managed and native honey bees.

No screened target was detected in the four *A*. *dorsata* or three *A*. *florea* colonies. This pattern does not reflect the full regional literature: DWV and BQCV have previously been detected in both species, and DWV has sometimes been reported less frequently than BQCV in wild *A*. *dorsata* and *A*. *florea* [31,32]. Their open-nesting or migratory ecology, differences in colony management and parasite associations, geographic and temporal exposure, and the small number sampled here could all influence detection patterns. Broader sampling is needed to determine which explanation applies in Cambodia and Laos.

### 4.2. Descriptive Differences Between Countries and Hosts

Detected viral richness and co-detection were higher in the Laos sample: five viral taxa (ABPV, BQCV, IAPV, LSV, and KBV) and four colonies with multiple viruses were recorded in Laos, compared with two viral taxa (ABPV and DWV-A) and one colony with multiple viruses in Cambodia. ABPV was the only virus shared between countries. These differences may reflect variation in host composition, management, geography, collection date, colony contact, or local environmental conditions.

Co-detections in Laos occurred in two *A*. *cerana* colonies and two *A*. *mellifera* colonies; the Cambodian co-detection occurred in *A*. *mellifera*. Because only three *A*. *mellifera* colonies were sampled in Laos, the pattern does not support a host-susceptibility comparison, but it shows that viral co-detection was not confined to one *Apis* species. Similar multi-host occurrence is biologically plausible where honey bee species overlap and use shared floral resources [23,31].

Seasonal changes in viral occurrence have been documented in *A*. *cerana* and *A*. *mellifera* [22]. The present country patterns may therefore provide hypotheses for seasonally replicated sampling, particularly for DWV-A in Cambodian *A*. *mellifera* and for the broader viral assemblage detected in Laos.

Complete colony-level management classifications were unavailable for the analytical dataset, so differences among managed, semi-managed, and wild colonies could not be evaluated statistically. Future surveys should record management intensity consistently to test whether it explains variation in pathogen richness or co-detection.

### 4.3. Fragment-Based Sequence Comparison and Regional Viral Affinities

Partial sequences confirmed the supported the identities of the detected viruses and *N*. *ceranae* and showed close similarity to previously reported Asian isolates. This indicates that the detected amplicons are related to pathogen variants already documented in the broader region and provides a molecular reference for future genomic surveys. Because each target was analyzed in a separate alignment, the trees describe sequence affinities within each amplified region rather than relationships among different viruses.

The ABPV, IAPV, and KBV results were interpreted independently despite the known relatedness of these viruses [33]. The short amplicons provide robust target confirmation but not the resolution needed for detailed phylogeographic reconstruction; longer genomic sequences will be required to evaluate finer-scale lineage structure.

### 4.4. Study Limitations and Future Directions

The study has five principal limitations. First, host sample sizes were uneven, particularly for *A*. *dorsata* and *A*. *florea*. Second, sampling covered a limited number of dates and was not designed to characterize seasonal variation. Third, standardized colony-health, colony-strength, management, and *V*. *destructor* infestation data were unavailable, preventing evaluation of clinical or mite-associated patterns. Fourth, the targeted panel cannot represent the full pathogen community. Fifth, conventional RT-PCR generated qualitative presence–absence data; low-copy infections may have remained below assay detection limits, and viral loads could not be estimated. Future work could combine balanced and seasonally replicated host sampling with standardized colony and mite assessments, complete management metadata, validated RT-qPCR assays, and longer genomic sequencing.

## 5. Conclusions

This study provides a targeted baseline survey of selected honey bee-associated viruses and *Nosema ceranae* in colonies sampled across multiple sites in Cambodia and Laos. ABPV, BQCV, DWV-A, IAPV, LSV, KBV, and *N*. *ceranae* were detected among the sampled *A*. *cerana* and *A*. *mellifera* colonies, generally at low detection frequencies in this dataset. No screened targets were detected in the small number of sampled *A*. *dorsata* and *A*. *florea* colonies; however, limited sample sizes and restricted temporal coverage preclude inference of pathogen absence in these species. Sequencing of PCR amplicons confirmed target identity and demonstrated similarity to previously reported reference sequences, supporting broad regional viral affinities. Because analyses were based on partial gene fragments, phylogenetic results are interpreted conservatively and are not used to infer transmission routes or epidemiological dynamics. Expanded and longitudinal surveys will be required to more comprehensively characterize pathogen occurrence and diversity among honey bee populations in the Mekong region.

## Data Availability

The data presented in this study are available upon request from the corresponding author.

## Supplementary Materials

The following supporting information can be downloaded at https://www.mdpi.com/article/10.3390/insects17090956/s1. Table S1: Sampling locations, collection dates, geographic coordinates, and numbers of colonies of four *Apis* species sampled at 10 sites in Cambodia and Laos; Table S2: Oligonucleotide primers used for PCR screening of RNA viruses and DNA-based pathogens and for amplification of β-actin as an internal control; Table S3: GenBank accession numbers and associated sample information for deposited mitochondrial cytochrome c oxidase subunit I (COI) barcode sequences and representative partial nucleotide sequences of six honey bee viruses and *Nosema ceranae* obtained from *Apis* colonies sampled in Cambodia and Laos; Table S4: Colony-level detection results for 10 RNA viruses and six DNA-based pathogens screened via PCR or RT-PCR in 53 *Apis* colonies sampled in Cambodia and Laos.
