# Supplementary material for: Baseline Detection of Honey Bee Viruses and Nosema ceranae in Apis Species from Cambodia and Laos
Source: Insects. 2026 Sep 14;17(9):956. doi: 10.3390/insects17090956 (PMC13607233; doi:10.3390/insects17090956)
Supplement: Supplementary file 1 [file insects-17-00956-s001.zip › insects-4425895-supplementary.pdf]

Supplementary

Table S1. Sampling locations, collection dates, geographic coordinates, and numbers of colonies of four *Apis* species sampled at 10 sites in Cambodia and Laos.

**Site No.**	**Location**	**Country**	**Collection date**	**Latitude (°N)**	**Longitude (°E)**	**Number of honey bee colony**				**Total colonies**
						A. cerana	***A. dorsata***	***A. florea***	***A. mellifera***	
1	Sisophon	Cambodia	May 2022	13.5789	102.9999	2	0	0	5	7
2	Battambang		May 2022	13.0834	103.1404	3	0	0	0	3
3	Siem Reap		May 2022	13.3489	103.7754	2	3	2	3	10
4	Phnom Penh		May 2022	11.5582	104.8142	0	0	0	4	4
5	Ratanakiri		July 2023	13.5971	106.9798	3	0	0	3	6
					**Total**	**10**	**3**	**2**	**15**	**30**
6	Oudomxay	Laos	May 2022	20.6861	101.9808	7	0	0	0	7
7	Vientiane		March 2023	17.9168	102.6318	3	1	1	0	5
8	Luang Prabang		March 2023	19.884	102.1435	0	0	0	3	3
9	Nabong		March 2023	18.0363	102.6357	6	0	0	0	6
10	Thong Pong		March 2023	17.9936	102.5358	2	0	0	0	2
					**Total**	**18**	**1**	**1**	**3**	**23**
					**Grand total**	**28**	**4**	**3**	**18**	**53**

Table S2 Oligonucleotide primers used for PCR screening of RNA viruses and DNA-based pathogens and for amplification of β-actin as an internal control.

Target	Primer name	Sequence (5'-3')	Size (bp)	Reference
Acute bee paralysis virus	ABPV-F	CTCAAGTTATACGTAAAATAGCTGGAATT	646	Gauthier et al., 2007
	ABPV-R	AACCAACCTTGCTTCCCTTTA		
Black queen cell virus	BQCV-F	TGGTCAGCTCCCACTACCTTAAAC	700	Benjeddou et al., 2001
	BQCV-R	GCAACAAGAAGAAACGTAAACCAC		
Chronic bee paralysis virus	CBPV-F	AGTTGTCATGGTTAACAGGATACGAG	455	Ribière et al., 2002
	CBPV-R	TCTAATCTTAGCACGAAAGCCGAG		
Deformed wing virus	DWV-F	ATCAGCGCTTAGTGGAGGAA	702	Chen et al., 2004
	DWV-R	TCGACAATTTTCGGACATCA		
Deformed wing virus type B	DWV_B-F	CATGGAAATGGGATCAAACC	722	Zioni et al., 2011
	DWV_B-R	CTTCCAAGGGCTCATCCATA		
Israeli acute paralysis virus	IAPV-F	GAAGGTTTGGGARGCYCCAYTWTGTAT	706	Yang et al., 2013
	IAPV-R	TGTTTGCRTCGGCHGTWGTTCCWGCAA		
Lake Sinai virus	LSV-F	GCCWCGRYTGTTGGTYCCCCC	578	Ravoet et al., 2013
	LSV-R	GAGGTGGCGGCGCSAGATAAAGT		
Kashmir bee virus	KBV-F	GATGAACGTCGACCTATTGA	415	Stoltz et al., 1995
	KBV-R	TGTGGGTTGGCTATGAGTCA		
Sacbrood virus	SBV-F	GCTGAGGTAGGATCTTTGCGT	824	Chen et al., 2004
	SBV-R	TCATCATCTTCACCATCCGA		
Slow bee paralysis virus	SBPV-F	GAGATGGATMGRCCTGAAGG	915	Manley et al., 2017
	SBPV-R	CATGAGCCCAKGARTGTGAA		
Nosema apis	N. apis F	CCATTGCCGGATAAGAGAGT	401	Chen et al., 2009
	N. apis R	CACGCATTGCTGCATCATTGAC		
Nosema bombi	Nbombi-SSU-Jf1	CCATGCATGTTTTTGAAGATTATTAT	323	Klee et al., 2006
	Nbombi-SSU-Jr1	CATATATTTTTAAAATATGAAACAATAA		
Nosema ceranae	SSUrRNA-f1	CACCAGGTTGATTCTGCCT	540	Tay et al., 2005
	SSUrRNA-r1b	TGTTCGTCCAGTCAGGGTCGTCA		
Paenibacillus larvae	AFB-F	CTTGTGTTTCTTTCGGGAGACGCCA	1,101	Dobbelaere et al., 2001
	AFB-R	TCTTAGAGTGCCCACCTCTGCG		

Ascosphaera apis	ITS primers 3-F1	TGTCTGTGCGGCTAGGTG	500	James & Skinner, 2005
	AscoAll2	GAWCACGACGCCGTCACT		
Crithidia bombi	Crith-F	GGAAACCACGGAATCACATAGACC	500	Li et al., 2012
	Crith-R	AGGAAGCCAAGTCATCCATCGC		
β-actin	β-actin-F	TTGTATGCCAACACTGTCCTTT	271	Simone et al., 2009
	β-actin-R	TGGCGCGATGATCTTAATTT		

Table S3 GenBank accession numbers and associated sample information for deposited mitochondrial cytochrome c oxidase subunit I (COI) barcode sequences and representative partial nucleotide sequences of six honey bee viruses and *Nosema ceranae* obtained from *Apis* colonies sampled in Cambodia and Laos.

No.	Target	Sample ID	Host species	Location	GenBank Accession number
1	mitochondrial cytochrome oxidase subunit 1	AC-SSP01	*A. cerana*	Cambodia	OP132592
2	mitochondrial cytochrome oxidase subunit 1	AC-SSP02	*A. cerana*	Cambodia	OP132593
3	mitochondrial cytochrome oxidase subunit 1	AM-SSP01	*A. mellifera*	Cambodia	OP132594
4	mitochondrial cytochrome oxidase subunit 1	AM-SSP02	*A. mellifera*	Cambodia	OP132595
5	mitochondrial cytochrome oxidase subunit 1	AM-SSP03	*A. mellifera*	Cambodia	OP132596
6	mitochondrial cytochrome oxidase subunit 1	AM-SSP04	*A. mellifera*	Cambodia	OP132597
7	mitochondrial cytochrome oxidase subunit 1	AM-SSP05	*A. mellifera*	Cambodia	OP132598
8	mitochondrial cytochrome oxidase subunit 1	AC-BTB01	*A. cerana*	Cambodia	OP132599
9	mitochondrial cytochrome oxidase subunit 1	AC-BTB02	*A. cerana*	Cambodia	OP132600
10	mitochondrial cytochrome oxidase subunit 1	AC-BTB03	*A. cerana*	Cambodia	OP132601
11	mitochondrial cytochrome oxidase subunit 1	AD-SR01	*A. dorsata*	Cambodia	OP132602
12	mitochondrial cytochrome oxidase subunit 1	AD-SR02	*A. dorsata*	Cambodia	OP132603
13	mitochondrial cytochrome oxidase subunit 1	AD-SR03	*A. dorsata*	Cambodia	OP132604
14	mitochondrial cytochrome oxidase subunit 1	AC-SR01	*A. cerana*	Cambodia	OP132605
15	mitochondrial cytochrome oxidase subunit 1	AC-SR02	*A. cerana*	Cambodia	OP132606
16	mitochondrial cytochrome oxidase subunit 1	AF-SR01	*A. florea*	Cambodia	OP132607
17	mitochondrial cytochrome oxidase subunit 1	AF-SR02	*A. florea*	Cambodia	OP132608
18	mitochondrial cytochrome oxidase subunit 1	AM-SR01	*A. mellifera*	Cambodia	OP132609
19	mitochondrial cytochrome oxidase subunit 1	AM-SR02	*A. mellifera*	Cambodia	OP132610
20	mitochondrial cytochrome oxidase subunit 1	AM-SR03	*A. mellifera*	Cambodia	OP132611

21	mitochondrial cytochrome oxidase subunit 1	AM-PP01	*A. mellifera*	Cambodia	OP132612
22	mitochondrial cytochrome oxidase subunit 1	AM-PP02	*A. mellifera*	Cambodia	OP132613
23	mitochondrial cytochrome oxidase subunit 1	AM-PP03	*A. mellifera*	Cambodia	OP132614
24	mitochondrial cytochrome oxidase subunit 1	AM-PP04	*A. mellifera*	Cambodia	OP132615
25	*N. ceranae*	AC-SR01	*A. cerana*	Cambodia	PP126485
26	*N. ceranae*	AM-SR02	*A. mellifera*	Cambodia	PP126486
27	*N. ceranae*	CBAM3	*A. mellifera*	Cambodia	PP126487
28	mitochondrial cytochrome oxidase subunit 1	AC-OX01	*A. cerana*	Laos	OP720954
29	mitochondrial cytochrome oxidase subunit 1	AC-OX02	*A. cerana*	Laos	OP720955
30	mitochondrial cytochrome oxidase subunit 1	AC-OX03	*A. cerana*	Laos	OP720956
31	mitochondrial cytochrome oxidase subunit 1	AC-OX04	*A. cerana*	Laos	OP720957
32	mitochondrial cytochrome oxidase subunit 1	AC-OX05	*A. cerana*	Laos	OP720958
33	mitochondrial cytochrome oxidase subunit 1	AC-OX06	*A. cerana*	Laos	OP720959
34	mitochondrial cytochrome oxidase subunit 1	AC-OX07	*A. cerana*	Laos	OP720960
35	mitochondrial cytochrome oxidase subunit 1	AC-WC8	*A. cerana*	Laos	OP720961
36	mitochondrial cytochrome oxidase subunit 1	AC-WC9	*A. cerana*	Laos	OP720962
37	mitochondrial cytochrome oxidase subunit 1	AD-SP10	*A. dorsata*	Laos	OP720963
38	mitochondrial cytochrome oxidase subunit 1	AC-SP11	*A. cerana*	Laos	OP720964
39	mitochondrial cytochrome oxidase subunit 1	AM-LP01	*A. mellifera*	Laos	OP720965
40	mitochondrial cytochrome oxidase subunit 1	AM-LP02	*A. mellifera*	Laos	OP720966
41	mitochondrial cytochrome oxidase subunit 1	AM-LP03	*A. mellifera*	Laos	OP720967
42	mitochondrial cytochrome oxidase subunit 1	NB-AC1	*A. cerana*	Laos	OR267449
43	mitochondrial cytochrome oxidase subunit 1	NB-AC2	*A. cerana*	Laos	OR267450
44	mitochondrial cytochrome oxidase subunit 1	NB-AC3	*A. cerana*	Laos	OR267451

45	mitochondrial cytochrome oxidase subunit 1	NB-AC4	*A. cerana*	Laos	OR267452
46	mitochondrial cytochrome oxidase subunit 1	NB-AC5	*A. cerana*	Laos	OR267453
47	mitochondrial cytochrome oxidase subunit 1	NB-AC6	*A. cerana*	Laos	OR267454
48	mitochondrial cytochrome oxidase subunit 1	TP-AC1	*A. cerana*	Laos	OR267455
49	mitochondrial cytochrome oxidase subunit 1	TP-AC2	*A. cerana*	Laos	OR267456
50	mitochondrial cytochrome oxidase subunit 1	LAF1	*A. florea*	Laos	OR267457
51	ABPV	PP-AM3	*A. mellifera*	Cambodia	OP896064
52	ABPV	OX6	*A. cerana*	Laos	OP896065
53	ABPV	WC8	*A. cerana*	Laos	OP896066
54	BQCV	LPAM1	*A. mellifera*	Laos	OP896067
55	BQCV	LPAM2	*A. mellifera*	Laos	OP896068
56	BQCV	LPAM3	*A. mellifera*	Laos	OP896069
57	DWV-A	PP-AM4	*A. mellifera*	Cambodia	OP896070
58	DWV-A	PP-AM3	*A. mellifera*	Cambodia	OP896071
59	DWV-A	PP-AM1	*A. mellifera*	Cambodia	OP896072
60	IAPV	OX6	*A. cerana*	Laos	OP896073
61	IAPV	WC8	*A. cerana*	Laos	OP896074
62	LSV	LPAM3	*A. mellifera*	Laos	OP896075
63	LSV	LPAM2	*A. mellifera*	Laos	OP896076
64	KBV	OX6	*A. cerana*	Laos	OQ200494

Table S4 Colony-level detection results for 10 RNA viruses and six DNA-based pathogens screened by PCR or RT-PCR in 53 *Apis* colonies sampled in Cambodia and Laos.

No.	Species	Location	Country	Code	Viruses										*P. larvae*	*N. apis*	*N. ceranae*	*N. bombi*	*C. bombi*	*A. apis*
					ABPV	BQCV	CBPV	DWV-A	DWV-B	IAPV	LSV	KBV	SBV	SBPV						
1	*A. cerana*	Sisophon	Cambodia	SS-AC1	ND.	ND.	ND.	ND.	ND.	ND.	ND.	ND.	ND.	ND.	ND.	ND.	ND.	ND.	ND.	ND.
2	*A. cerana*			SS-AC2	ND.	ND.	ND.	ND.	ND.	ND.	ND.	ND.	ND.	ND.	ND.	ND.	ND.	ND.	ND.	ND.
3	*A. mellifera*			SS-AM1	ND.	ND.	ND.	ND.	ND.	ND.	ND.	ND.	ND.	ND.	ND.	ND.	ND.	ND.	ND.	ND.
4	*A. mellifera*			SS-AM2	ND.	ND.	ND.	ND.	ND.	ND.	ND.	ND.	ND.	ND.	ND.	ND.	ND.	ND.	ND.	ND.
5	*A. mellifera*			SS-AM3	ND.	ND.	ND.	ND.	ND.	ND.	ND.	ND.	ND.	ND.	ND.	ND.	ND.	ND.	ND.	ND.
6	*A. mellifera*			SS-AM4	ND.	ND.	ND.	ND.	ND.	ND.	ND.	ND.	ND.	ND.	ND.	ND.	ND.	ND.	ND.	ND.
7	*A. mellifera*			SS-AM5	ND.	ND.	ND.	ND.	ND.	ND.	ND.	ND.	ND.	ND.	ND.	ND.	ND.	ND.	ND.	ND.
8	*A. cerana*	Battambang		BTB-AC1	ND.	ND.	ND.	ND.	ND.	ND.	ND.	ND.	ND.	ND.	ND.	ND.	ND.	ND.	ND.	ND.

9	*A. cerana*			BTB-AC2	ND.	ND.	ND.	ND.	ND.	ND.	ND.	ND.	ND.	ND.	ND.	ND.	ND.	ND.	ND.	ND.
10	*A. cerana*			BTB-AC3	ND.	ND.	ND.	ND.	ND.	ND.	ND.	ND.	ND.	ND.	ND.	ND.	ND.	ND.	ND.	ND.
11	*A. dorsata*	Siem Reap		SR-AD1	ND.	ND.	ND.	ND.	ND.	ND.	ND.	ND.	ND.	ND.	ND.	ND.	ND.	ND.	ND.	ND.
12	*A. dorsata*			SR-AD2	ND.	ND.	ND.	ND.	ND.	ND.	ND.	ND.	ND.	ND.	ND.	ND.	ND.	ND.	ND.	ND.
13	*A. dorsata*			SR-AD3	ND.	ND.	ND.	ND.	ND.	ND.	ND.	ND.	ND.	ND.	ND.	ND.	ND.	ND.	ND.	ND.
14	*A. florea*			SR-AF1	ND.	ND.	ND.	ND.	ND.	ND.	ND.	ND.	ND.	ND.	ND.	ND.	ND.	ND.	ND.	ND.
15	*A. florea*			SR-AF2	ND.	ND.	ND.	ND.	ND.	ND.	ND.	ND.	ND.	ND.	ND.	ND.	ND.	ND.	ND.	ND.
16	*A. mellifera*			SR-AM1	ND.	ND.	ND.	ND.	ND.	ND.	ND.	ND.	ND.	ND.	ND.	ND.	ND.	ND.	ND.	ND.
17	*A. mellifera*			SR-AM2	ND.	ND.	ND.	ND.	ND.	ND.	ND.	ND.	ND.	ND.	ND.	ND.	+	ND.	ND.	ND.
18	*A. mellifera*			SR-AM3	ND.	ND.	ND.	ND.	ND.	ND.	ND.	ND.	ND.	ND.	ND.	ND.	ND.	ND.	ND.	ND.
19	*A. cerana*			SR-AC1	ND.	ND.	ND.	ND.	ND.	ND.	ND.	ND.	ND.	ND.	ND.	ND.	+	ND.	ND.	ND.

20	*A. cerana*			SR-AC2	ND.	ND.	ND.	ND.	ND.	ND.	ND.	ND.	ND.	ND.	ND.	ND.	ND.	ND.	ND.	ND.
21	*A. mellifera*	Phnom Penh		PP-AM1	ND.	ND.	ND.	+	ND.	ND.	ND.	ND.	ND.	ND.	ND.	ND.	ND.	ND.	ND.	ND.
22	*A. mellifera*			PP-AM2	ND.	ND.	ND.	ND.	ND.	ND.	ND.	ND.	ND.	ND.	ND.	ND.	ND.	ND.	ND.	ND.
23	*A. mellifera*			PP-AM3	+	ND.	ND.	+	ND.	ND.	ND.	ND.	ND.	ND.	ND.	ND.	ND.	ND.	ND.	ND.
24	*A. mellifera*			PP-AM4	ND.	ND.	ND.	+	ND.	ND.	ND.	ND.	ND.	ND.	ND.	ND.	ND.	ND.	ND.	ND.
25	*A. cerana*	Ratanakiri		CBAC1	ND.	ND.	ND.	ND.	ND.	ND.	ND.	ND.	ND.	ND.	ND.	ND.	ND.	ND.	ND.	ND.
26	*A. cerana*			CBAC2	ND.	ND.	ND.	ND.	ND.	ND.	ND.	ND.	ND.	ND.	ND.	ND.	ND.	ND.	ND.	ND.
27	*A. cerana*			CBAC3	ND.	ND.	ND.	ND.	ND.	ND.	ND.	ND.	ND.	ND.	ND.	ND.	ND.	ND.	ND.	ND.
28	*A. mellifera*			CBAM1	ND.	ND.	ND.	ND.	ND.	ND.	ND.	ND.	ND.	ND.	ND.	ND.	ND.	ND.	ND.	ND.
29	*A. mellifera*			CBAM2	ND.	ND.	ND.	+	ND.	ND.	ND.	ND.	ND.	ND.	ND.	ND.	ND.	ND.	ND.	ND.
30	*A. mellifera*			CBAM3	ND.	ND.	ND.	ND.	ND.	ND.	ND.	ND.	ND.	ND.	ND.	ND.	+	ND.	ND.	ND.

31	*A. cerana*	Oudomxay	Laos	OX1	ND.	ND.	ND.	ND.	ND.	ND.	ND.	ND.	ND.	ND.	ND.	ND.	ND.	ND.	ND.	ND.
32	*A. cerana*			OX2	ND.	ND.	ND.	ND.	ND.	ND.	ND.	ND.	ND.	ND.	ND.	ND.	ND.	ND.	ND.	ND.
33	*A. cerana*			OX3	ND.	ND.	ND.	ND.	ND.	ND.	ND.	ND.	ND.	ND.	ND.	ND.	ND.	ND.	ND.	ND.
34	*A. cerana*			OX4	ND.	ND.	ND.	ND.	ND.	ND.	ND.	ND.	ND.	ND.	ND.	ND.	ND.	ND.	ND.	ND.
35	*A. cerana*			OX5	ND.	ND.	ND.	ND.	ND.	ND.	ND.	ND.	ND.	ND.	ND.	ND.	ND.	ND.	ND.	ND.
36	*A. cerana*			OX6	+	ND.	ND.	ND.	ND.	+	ND.	+	ND.	ND.	ND.	ND.	ND.	ND.	ND.	ND.
37	*A. cerana*			OX7	ND.	ND.	ND.	ND.	ND.	ND.	ND.	ND.	ND.	ND.	ND.	ND.	ND.	ND.	ND.	ND.
38	*A. cerana*	Vientiane		WC8	+	ND.	ND.	ND.	ND.	+	ND.	ND.	ND.	ND.	ND.	ND.	ND.	ND.	ND.	ND.
39	*A. cerana*			WC9	ND.	ND.	ND.	ND.	ND.	ND.	ND.	ND.	ND.	ND.	ND.	ND.	ND.	ND.	ND.	ND.
40	*A. dorsata*			SP10	ND.	ND.	ND.	ND.	ND.	ND.	ND.	ND.	ND.	ND.	ND.	ND.	ND.	ND.	ND.	ND.
41	*A. cerana*			SP11	ND.	ND.	ND.	ND.	ND.	ND.	ND.	ND.	ND.	ND.	ND.	ND.	ND.	ND.	ND.	ND.

42	*A. mellifera*	Luang Prabang		LPA M1	ND.	+	ND.	ND.	ND.	ND.	ND.	ND.	ND.	ND.	ND.	ND.	ND.	ND.	ND.	ND.
43	*A. mellifera*			LPA M2	ND.	+	ND.	ND.	ND.	ND.	+	ND.	ND.	ND.	ND.	ND.	ND.	ND.	ND.	ND.
44	*A. mellifera*			LPA M3	ND.	+	ND.	ND.	ND.	ND.	+	ND.	ND.	ND.	ND.	ND.	ND.	ND.	ND.	ND.
45	*A. cerana*	Nabong		NBA C1	ND.	ND.	ND.	ND.	ND.	ND.	ND.	ND.	ND.	ND.	ND.	ND.	ND.	ND.	ND.	ND.
46	*A. cerana*			NBA C2	ND.	ND.	ND.	ND.	ND.	ND.	ND.	ND.	ND.	ND.	ND.	ND.	ND.	ND.	ND.	ND.
47	*A. cerana*			NBA C3	ND.	ND.	ND.	ND.	ND.	ND.	ND.	ND.	ND.	ND.	ND.	ND.	ND.	ND.	ND.	ND.
48	*A. cerana*			NBA C4	ND.	ND.	ND.	ND.	ND.	ND.	ND.	ND.	ND.	ND.	ND.	ND.	ND.	ND.	ND.	ND.
49	*A. cerana*			NBA C5	ND.	ND.	ND.	ND.	ND.	ND.	ND.	ND.	ND.	ND.	ND.	ND.	ND.	ND.	ND.	ND.
50	*A. cerana*			NBA C6	ND.	ND.	ND.	ND.	ND.	ND.	ND.	ND.	ND.	ND.	ND.	ND.	ND.	ND.	ND.	ND.
51	*A. cerana*	Thong Pong		TPA C1	ND.	ND.	ND.	ND.	ND.	ND.	ND.	ND.	ND.	ND.	ND.	ND.	ND.	ND.	ND.	ND.
52	*A. cerana*			TPA C2	ND.	ND.	ND.	ND.	ND.	ND.	ND.	ND.	ND.	ND.	ND.	ND.	ND.	ND.	ND.	ND.

53	*A. florea*	Vientiane		LAF1	ND.	ND.	ND.	ND.	ND.	ND.	ND.	ND.	ND.	ND.	ND.	ND.	ND.	ND.	ND.	ND.

Note: ND., not detected; +, detected.
